# Group early intervention eye movement desensitization and reprocessing therapy as a video-conference psychotherapy with frontline/emergency workers in response to the COVID-19 pandemic in the treatment of post-traumatic stress disorder and moral injury—An RCT study

**DOI:** 10.3389/fpsyg.2023.1129912

**Published:** 2023-03-23

**Authors:** Derek Farrell, Johnny Moran, Zeynep Zat, Paul W. Miller, Lorraine Knibbs, Penny Papanikolopoulos, Tessa Prattos, Iain McGowan, Derek McLaughlin, Ian Barron, Cordula Mattheß, Matthew D. Kiernan

**Affiliations:** ^1^Department for Violence Prevention, Trauma and Criminology (VPTC), School of Psychology, University of Worcester, Worcester, United Kingdom; ^2^School of Nursing and Midwifery, Queen’s University, Belfast, Northern Ireland, United Kingdom; ^3^School of Nursing, Magee Campus, Ulster University, Northern Ireland, United Kingdom; ^4^Centre for International Education, College of Education, University of Massachusetts, Amherst, MA, United States; ^5^Northern Hub for Veteran and Military Families’ Research, Northumbria University, Newcastle upon Tyne, United Kingdom

**Keywords:** EMDR early intervention, group treatment, COVID-19, emergency and frontline workers, therapist rotation, posttraumatic stress disorder, moral injury

## Abstract

**Objective:**

Frontline mental health, emergency, law enforcement, and social workers have faced unprecedented psychological distress in responding to the COVID-19 pandemic. The purpose of the RCT (Randomized Controls Trial) study was to investigate the effectiveness of a Group EMDR (Eye Movement Desensitization and Reprocessing) therapy (Group Traumatic Episode Protocol—GTEP) in the treatment of Post-Traumatic Stress Disorder (PTSD) and Moral Injury. The treatment focus is an early intervention, group trauma treatment, delivered remotely as video-conference psychotherapy (VCP). This early intervention used an intensive treatment delivery of 4x2h sessions over 1-week. Additionally, the group EMDR intervention utilized therapist rotation in treatment delivery.

**Methods:**

The study’s design comprised a delayed (1-month) treatment intervention (control) versus an active group. Measurements included the International Trauma Questionnaire (ITQ), Generalized Anxiety Disorder Assessment (GAD-7), Patient Health Questionnaire (PHQ-9), Moral Injury Events Scale (MIES), and a Quality-of-Life psychometric (EQ-5D), tested at T0, T1: pre—treatment, T2: post-treatment, T3: 1-month follow-up (FU), T4: 3-month FU, and T5: 6-month FU. The Adverse Childhood Experiences – International version (ACEs), Benevolent Childhood Experience (BCEs) was ascertained at pre-treatment only. *N* = 85 completed the study.

**Results:**

Results highlight a significant treatment effect within both active and control groups. *Post Hoc* comparisons of the ITQ demonstrated a significant difference between T1 pre (mean 36.8, SD 14.8) and T2 post (21.2, 15.1) (t11.58) = 15.68, *p* < 0.001). Further changes were also seen related to co-morbid factors. *Post Hoc* comparisons of the GAD-7 demonstrated significant difference between T1 pre (11.2, 4.91) and T2 post (6.49, 4.73) (*t* = 6.22) = 4.41, *p* < 0.001; with significant difference also with the PHQ-9 between T1 pre (11.7, 5.68) and T2 post (6.64, 5.79) (*t* = 6.30) = 3.95, *p* < 0.001, *d* = 0.71. The treatment effect occurred irrespective of either ACEs/BCEs during childhood. However, regarding Moral Injury, the MIES demonstrated no treatment effect between T1 pre and T5 6-month FU. The study’s findings discuss the impact of Group EMDR therapy delivered remotely as video-conference psychotherapy (VCP) and the benefits of including a therapist/rotation model as a means of treatment delivery. However, despite promising results suggesting a large treatment effect in the treatment of trauma and adverse memories, including co-morbid symptoms, research results yielded no treatment effect in frontline/emergency workers in addressing moral injury related to the COVID-19 pandemic.

**Conclusion:**

The NICE (2018) guidance on PTSD highlighted the paucity of EMDR therapy research used as an early intervention. The primary rationale for this study was to address this critical issue. In summary, treatment results for group EMDR, delivered virtually, intensively, using therapist rotation are tentatively promising, however, the moral dimensions of trauma need consideration for future research, intervention development, and potential for further scalability. The data contributes to the emerging literature on early trauma interventions.

**Clinical Trial Registration:**Clinicaltrials.gov, ISRCTN16933691.

## Introduction

Frontline mental health, emergency, law enforcement, and social workers have faced unprecedented psychological distress in responding to the COVID-19 pandemic, with around 22% meeting the criteria for Post-Traumatic Stress Disorder [PTSD]–a mental health condition caused by a traumatic experience (Wild et al., 2016, 2022; Johnson et al., 2020; Greene et al., 2021; Nagarajan et al., 2022; Watson, 2022; Yunitri et al., 2022). Continued psychological distress potentially may result in adverse outcomes including substance misuse, suicide risk, burnout, compassion fatigue, and secondary traumatization (Arpacioglu et al., 2021; Hooper et al., 2021). Although the impact of psychological harm may have delayed onset, implementing effective early intervention measures that seek to mitigate the potential development of PTSD is essential (Haugen et al., 2012; Hooper et al., 2021; Brunet et al., 2022). Early intervention in the aftermath of psychological trauma is defined as a treatment administered at the earliest possible time, sometimes within hours of the traumatic event, but is considered any intervention within the first 3 months after exposure (Dyregrov and Regel, 2012) consider four important principles related to early trauma interventions: immediacy, proximity, expectancy, and flexibility. Acknowledging the COVID-19 pandemic has sharply increased the demand on frontline emergency workers; nonetheless, organizations require evidence-based information about available psychological programs to address the mental health needs of staff (Jecker et al., 2020; Rasheed et al., 2020; Sritharan et al., 2020; Beames et al., 2021; Billings et al., 2021; Do and Frank, 2021; Norman et al., 2021; Carmassi et al., 2022; Feingold et al., 2022; Ghahramani et al., 2022; Smith et al., 2022). COVID-19 risk factors on mental health depend on the individual and the context. These included the risk heightened stress and anxiety, limited resources and protection, exposure to the virus and fear of exposing family, partners, and relatives, long shift patterns, sleep disruption and deprivation, burnout, and broader impact on work/life balance (Johnson et al., 2020; Raudenská et al., 2020; Sasangohar et al., 2020; Shreffler et al., 2020; de Kock et al., 2021; Lasalvia et al., 2021). Additionally, pre-existing anxiety/depression, exposure to previous adverse life experiences, and dissociative symptoms may contribute to PTSD in frontline health and emergency workers (Greenberg et al., 2020; Williamson et al., 2020; Billings et al., 2021; Greene et al., 2021; Miguel-Puga et al., 2021).

Ruck et al. (2013) stipulate that early trauma interventions are designed to neither prevent, nor treat, PTSD. Early organization interventions are useful in facilitating mutual support, identifying those that may require additional assistance, improving social cohesion, reducing harmful responses, and improving occupational functioning (Creamer et al., 2012; Dyregrov and Regel, 2012; Richins et al., 2020). Bryant (2021, 2022) and Magruder et al. (2017) consider that despite the continued debate surrounding the optimal strategies used in the immediate period following trauma exposure, the specific objective should always be preventing the onset of PTSD and other co-morbidities.

The dynamic nature of PTSD in the immediate aftermath of trauma presents both opportunities and challenges concerning early trauma interventions (Price et al., 2018). Furthermore, the malleability of symptoms post-adversity exposure suggests that early interventions can alter the course of trauma sequelae, provided those interventions are both flexible and adaptive (Asmussen et al., 2019; Roberts et al., 2019; Hooper et al., 2021; Bryant, 2022). Therefore, the primary endeavor of any early trauma intervention response should be:

1. Reduce trauma stress reactions.
2. Provide authentic support.
3. Enhance coping.
4. Empower resilience.
5. Minimize the risk of burnout and vicarious traumatization.
6. Diminish the risk of developing other mental health and psychological difficulties with the potential to impact psychological well-being.

Roberts et al. (2019) published a systematic review of 61 studies of early psychological interventions. Their review supported trauma-focused cognitive behavioral therapy (CBT-TF), cognitive therapy without exposure, and eye movement desensitization and reprocessing (EMDR) for individuals reporting traumatic stress symptoms. They concluded that the research was more robust for CBT-TF. However, NICE guidance on PTSD (2018) highlights the lack of research supporting using EMDR as an early trauma intervention.

EMDR therapy is an empirically supported treatment for psychological trauma, endorsed by the World Health Organisation (2013), United Nations High Commission for Refugees (2022) and the International Society for Traumatic Stress Studies (2019). It is a psychological treatment for pathogenic (trauma) memories and their associated stress symptoms using a model of pathogenesis and change known as adaptive information processing (Hase et al., 2017; Hase, 2021; Farrell et al., 2022; Laliotis and Shapiro, 2022; Wippich et al., 2023). Shapiro (2017) considers EMDR to desensitize disturbing memories and stimulates the reprocessing of associated thoughts, feelings, and sensations towards adaptive resolution. Although a recent systematic review concluded EMDR is effective for first responders, they concurred with NICE (2018) in further indicating the quality of studies as weak or medium, highlighting several gaps and unanswered questions within the academic literature including high risk of bias, limited availability of data including safety and harm-related information (Oosterbaan et al., 2019; Bryant, 2021, 2022; Kaptan et al., 2021; Morris et al., 2022).

A humanitarian crisis emerges when an event threatens a population’s health, safety, and well-being. COVID-19 creates a familiar narrative frequently witnessed in humanitarian emergencies where demand for mental health provision outstrips the supply available (Carriere, 2014; Dunkley, 2018; Eichfeld et al., 2019; Mattheß et al., 2019, 2020; Farrell et al., 2020; Pupat et al., 2022). As Bryant (2022) highlighted earlier, interventions need adaptation to meet the needs of as many as possible. One such adaptation within EMDR therapy is Group Interventions. Kaptan et al. (2021) undertook a review of 22 studies using Group EMDR Interventions of which 13 studies examined the EMDR Integrative Group Treatment Protocol (IGTP), four studies the EMDR Group Traumatic Episode Protocol (G-TEP), four studies of EMDR Integrative Group Treatment Protocol for Ongoing Traumatic Stress and one study considered the EMDR Group Protocol with Children. Results suggested that Group EMDR protocols might effectively improve a wide range of mental health-related outcomes, including post-traumatic stress disorder (PTSD), depression and anxiety, compared with pre-treatment and control groups. However, Kaptan et al. (2021) concluded that the included studies are limited to methodological challenges with a high risk of bias.

The Group Trauma Episode Protocol [GTEP], developed by Shapiro and Laub (2008), is an evidence-based EMDR intervention used in the treatment of recent natural and human disasters (Acarturk et al., 2016; Roberts, 2018; Womersley and Arikut-Treece, 2019; Kaptan et al., 2021; Pink et al., 2022). An essential aspect of GTEP is that the client does not disclose any details regarding their trauma experiences. Within EMDR therapy this is known as ‘Blind 2 therapist (Farrell et al., 2020, 2022). There are distinct advantages to this. The rationale surrounding non-disclosure may potentially involve trauma memories that invoke shame, survivor guilt, moral injury, or fear of recrimination and stigma. Dimensions surrounding moral injury are pertinent as these either relates to inner core values, or external such as betrayal or breaches of trust.

For frontline and emergency workers, stigma remains a substantial barrier to seeking psychological support (Clement et al., 2015; Gronholm et al., 2021). Diop et al. (2022), in a study investigating 723 frontline workers, determined that 44% expressed concerns about being stigmatized and excluded from serving those affected by COVID-19, highlighting that stigma can be both internal and external. A study carried out in India with frontline healthcare workers in direct management of COVID-19 patients (Sachdeva et al., 2021) declared that 75% experienced self-stigma – primarily in the form of guilt in potentially exposing their families to the coronavirus.

**Figure 1:**
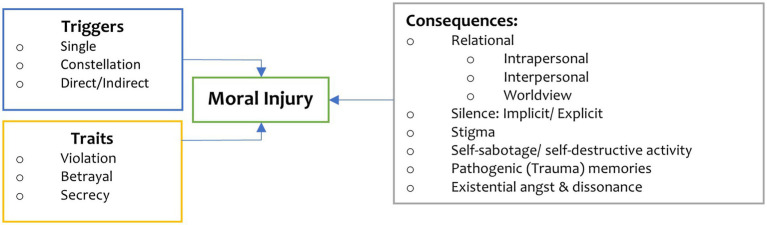
Key aspects of moral injury and COVID-19.

In addition to Acute Stress Disorder [ASD] (Brunet et al., 2022) and PTSD, another critical issue to return to, is moral injury. In reviewing the academic literature outlined in Figure 1, moral injury results from an act, or failure to act, that creates an ethical transgression, either by self or witnessed, which damages one’s conscience or moral compass (Litz et al., 2009) refers to the impact of moral injury as emotional, psychological, social, behavioral, and spiritual. However, there is insufficient explanation of moral injury within the current understanding of PTSD and its subsequent treatment (Barnes et al., 2019; Griffin et al., 2019; Koenig et al., 2019, 2020). Treating core PTSD symptoms does not address moral trauma—the same is true *vice-versa* (Farnsworth, 2019; Borges et al., 2020). A further definition of moral injury provided by Brock and Lettini (2012) describes it as a ‘wound of the soul’ when existing core moral foundations cannot be justified, sufficiently processed, and integrated into a reliable identity and meaning system sustains relationships and human flourishing. A study carried out with military mental health nurses by Jamieson et al. (2020a,b) relates moral injury to frontline workers. They describe it as experiencing existential, psychological, social, emotional, and spiritual/religious damage arising from a violation or betrayal (by omission or commission) of the core moral framework and manifesting through feelings of shame, guilt, stigma, and self-condemning, or self-sabotaging behaviors. The core aspects of moral injury pertaining to the COVID-19 pandemic are highlighted in Figure 1 (Koenig et al., 2019; Borges et al., 2020; Williamson et al., 2020; Amsalem et al., 2021; Cartolovni et al., 2021).

Implementing an early trauma intervention during COVID-19, and in government-enforced lockdown, presented several logistical challenges. Firstly, the public health management of COVID-19 involved social distancing to reduce risk of infection. One of the management strategies utilized to address this required a shift towards video-conferencing psychotherapy [VCP] (Crowe et al., 2020). As Healthcare providers closed their doors to face-to-face intervention, video-conferencing treatment platforms became, for many, the only viable option (Turgoose et al., 2018; Watts et al., 2020; Wind et al., 2020; Farrell et al., 2022). To date, several research studies indicate that psychological treatment, delivered remotely though video-conference platforms, are both safe and feasible. Furthermore, that they offer greater flexibility, and improve equity of access (Appleton et al., 2021; Broadbear et al., 2021; Cantone et al., 2021; Cataldo et al., 2021; Dharwadkar et al., 2021; Oudshoorn et al., 2021; Puspitasari et al., 2021; Shklarski et al., 2021a,b; Vera San Juan et al., 2021; Milosevic et al., 2022). Smith et al. (2022), however, considers that although the evidence-base is promising further interrogation and research is needed to further explore the ethical and moral dimensions of video-conference psychotherapy.

According to Herman and Kallivayalil (2018), outcome research supports group interventions in the treatment of PTSD; however, they conclude that there is limited evidence to support the superiority of one group model over another. The research evidence exploring group trauma treatment for PTSD using VCP is limited, especially regarding COVID-19. One group trauma treatment approach is Eye Movement Desensitization and Reprocessing (EMDR). Most current research demonstrates its effectiveness with Post-Traumatic Stress Disorder [PTSD] and Complex PTSD. However, EMDR therapy has been used extensively as an early intervention as part of trauma capacity building throughout the world (Farrell et al., 2011, 2013, 2020; Eichfeld et al., 2019; Mattheß et al., 2019, 2020; Covers et al., 2021; Tarquinio et al., 2021). Table 1 highlights the advantages and disadvantages of using EMDR therapy as an early intervention using virtual platforms.

**Table 1:** Advantages and disadvantages of early intervention EMDR therapy using visual platforms.

Advantages	Disadvantages
*o Brief intervention for the purpose of trauma symptom reduction* *o System of triage/ risk assessment in environments of limited resources* *o Provides trauma informed psychoeducation* *o Implementation of trauma informed stress regulation strategies* *o Distinct trauma confrontation intervention* *o Empowers individual and community resilience* *o Potential for implementation and delivery on consecutive days (intensive treatment)* *o Health economic benefits* *o Potential for ‘tasking-shifting’ to allied/ non-mental health workers* *o Enables specific teaching and learning in a focused trauma treatment intervention* *o Greater flexibility, logistical, linguistic, and cultural adaptability* *o Promotes re-invention, resilience, and post-traumatic growth*	*o Access to technology and software* *o Unreliable technology and functioning* *o Concerns about privacy and confidentiality* *o Often dependent on access to Wi-Fi and power supply* *o Difficulty to respond in crisis situations* *o Not appropriate for serious mental health problems and concerns* *o Concerns about risk* *o Restricts forms of non-verbal communication* *o Ethical and legal concerns of carrying out treatment with clients from different geographical areas/ regions*

Replicating an innovative approach used in the treatment of PTSD (van Minnen et al., 2018) utilized therapist rotation in delivering the treatment intervention. In this contemporary approach Trauma Therapists rotate between patients during the treatment intervention. As Table 2 accentuates, there are several distinct advantages and disadvantages to using therapist rotation (Krampe and Ehrenreich, 2012; van Minnen et al., 2018):

**Table 2:** Advantages and disadvantages of therapist rotation model in EMDR therapy.

Advantages	Disadvantages
*o Reducing interpersonal dependency on individual therapists* *o Reducing trauma therapists’ anxiety regarding trauma confrontation (exposure)* *o Improves ‘patient-focused’ intervention* *o Increased confidence in carrying out trauma-focused protocolized interventions* *o Increases trauma treatment fidelity* *o Greater cogency in overall patient treatment plan* *o Improving confidence in directly challenging trauma avoidance behaviors* *o Reduces the risk of vicarious traumatization of trauma therapists*	*o Reliant on effective communication and handover between team members* *o Potential divergence in confidence and ability in running treatment sessions* *o Requires strong team adhesion, team support and robust clinical supervision* *o Requires ‘buy in’ from clients and active client engagement* *o Client’s may consider the intervention as not bespoke to their needs* *o Focus of the intervention does not address individual issues outside the remit of the study* *o Difficult to research and evaluate the dyadic relationship*

As Kaptan et al. (2021) emphasized, more trauma-focused clinical trials, especially early intervention, incorporating robust methodology, larger sample sizes with participants who meet clear diagnostic criteria and including necessary follow-up data is essential. Addressing these salient issues was the primary driver in the inception of this COVID-19 randomized-control clinical trial. Mindful of the issues raised so far, this clinical trial wished to explore the following: early trauma intervention, use of video-conference psychotherapy, group treatment, Blind-2-therapist, intensive treatment, and therapist rotation – with a primary treatment target group Emergency Workers on the frontline of the COVID-19 pandemic who demonstrated trauma sequelae.

The broad research objectives for the study were:

1. Is Early Intervention EMDR Video Group Therapy (VGTEP) a safe, and efficient treatment intervention for Frontline/Emergency/Keyworkers, who experience psychological trauma in response to COVID-19 with regards to trauma sequalae and co-morbid symptoms.
2. Is Early Intervention EMDR Video Group Therapy (VGTEP) a relevant and effective treatment intervention for Frontline/Emergency/Keyworkers, who experience psychological trauma in response to COVID-19 with regards to both recruitment and retention to the study?

## Materials and methods

### Design

The University of Worcester (United Kingdom) granted ethical approval for the study [CBPS19200030]. The research study strictly adhered to the approval granted. Additionally, the study adhered to the British Psychological Society (BPS) Guidelines on ‘Conducting Research with Human Participants during COVID-19 (2020). The study registered as a clinical trial ID ISRCTN16933691 (2020) and hosted by the Trauma Response Network (TRN) – Ireland, an NGO organization offering early trauma interventions. Research participants recruited *via* a social networking and advertising strategy coordinated by TRN Ireland, which involved media outlets and radio stations in Ireland, Northern Ireland, and Great Britain. Interest in participation into the study was such that recruitment, although from the Island of Ireland, also included international participants from United States, Canada, Australia, New Zealand, Greece, and Turkey. The treatment intervention used for the clinical trial was the EMDR Group Traumatic Episode Protocol – version 9 (Shapiro, 2019–unpublished) used as a video-conference psychotherapy (VGTEP).

A Randomized Control Trial (RCT) Delayed Start Design used for the clinical study compared an active treatment intervention versus a control group (delayed treatment – 1 month) using the same intervention. The clinical trial conducted from July 2020 – March 2022 during the primary lockdown periods. The RCT consisted of two cohorts:

o Active Cohort 1: EMDR therapy VGTEP Treatment (4 sessions of approximately 2 h duration which equated to 8 h total treatment intervention) implemented as an intensive intervention for 1 week.
o Control Cohort 2: 4 weeks Delayed intervention of EMDR therapy VGTEP. Treatment involved an intensive intervention within 1 week.

The study incorporated six data points including T0 (control group), T1 (pre), T2 (post), T3 (1-month FU), T4 (3-month FU), and T5 (6-month FU) (Marcus et al., 2004; Ostacoli et al., 2018). All included research clients provided written consent before enrolment into the clinical trial.

The RCT generates six hypotheses to determine that the Virtual EMDR Group Intervention was safe, relevant, effective, and efficient:

*Hypothesis 1*: There is no difference between active and control (delayed) groups regarding trauma sequelae—measured with the International Trauma Questionnaire (ITQ) (Cloitre et al., 2018) when comparing T1, T2, T3, T4, and T5.

*Hypothesis 2*: There is no difference between active and control (delayed) groups regarding co-morbid sequelae – measured with the Patient Health Questionnaire (PHQ-9) (Kroenke et al., 2001) and the Generalized Anxiety Disorder (GAD-7) (Williams, 2014) over the T1-T5 time periods.

*Hypothesis 3*: Treating the trauma sequelae will reduce the level of moral injury as demonstrated with the Moral Injury Events Scale (MIES) ((Nash et al., 2013), demonstrated between T1 and T5, for both active and control groups.

*Hypothesis 4*: Using Trauma-Focused Therapist rotation will demonstrate no change in clinical diagnosis between T1 and T5, for both active and control groups.

*Hypothesis 5*: A negative correlation exists, within the target population, between Adverse Childhood Experiences (ACEs) and Benevolent Childhood Experiences (BCEs) (Narayan et al., 2018) regarding evidence supporting resilience and posttraumatic growth.

*Hypothesis 6*: There is no difference between active and control groups regarding quality of life using the EQ-5D – generic measure of an individual’s health status when comparing between T1 and T5.

### Participants

The clients used for this clinical trial were Frontline Health and Social Care workers or Emergency/First Responders directly working on the frontline of the COVID-19 pandemic utilizing the following inclusion/exclusion criteria:

Inclusion criteria:

o Adults (18 years and above).
o Front Line Health, Emergency and Social Care Workers experiencing Psychological Distress and Trauma in response to frontline working addressing the COVID-19 virus.
o Currently in active employment.
o Symptoms indicative of psychological distress and impact of psychological well-being and functioning measured using the Impact of Events Scale Revised Score of 24 and above.

Exclusion criteria:

o Non-trauma exposure.
o Impact of Events Scale Revised score of 23 or below.
o Undergoing current treatment (physical and/or psychological).
o Present evidence of psychosis.

As mentioned earlier as this was a Republic of Ireland Research initiative, research participants were recruited either from the Island of Ireland or internationally. A recruitment initiative was carried out utilizing media organizations within the Republic of Ireland. Recruitment to participate in the clinical trial used self-selection access through the Trauma Response Network (TRN) Ireland website through a secure platform. Each participant was also assessed by TRN Ireland administrative staff, who provided further information about the study outlining what participation involved. Those wishing to participate were provided with a detailed, research participant information sheet and research consent form.

The Impact of Events Scale–Revised (IES-R; Weiss and Marmar, 1997) was the primary screening tool used for the purpose of recruitment only. The IES-R has been tested for factor structure, internal consistency, concurrent validity, and discriminative validity by Beck et al. (2008). An IES-R score of 24 was used as the threshold for entering the study. The IES-R is an internationally recognized psychometric tool to assist in a diagnosis of Post-Traumatic Stress Disorder. The IES-R consists of 22 questions and provides four scores: total, intrusion, avoidance, and hyperarousal. The scoring is a maximum of 88. Asukai et al. (2002) stipulate that an IES-R score of 24–32 indicates clinical concern of either partial PTSD or at least some of its symptoms. Additionally (Creamer et al., 2003) advises that scores between 33 and 38 represent a cut-off score for a probable diagnosis of PTSD. A score of 39 and above is enough to suppress the immune system’s functioning (Izutsu et al., 2004, 2008).

Mirabilis Health Institute, Belfast, Northern Ireland, provided medical supervision for the clinical trial and was responsible for clinical risk assessment and triage.

**Figure 2:**
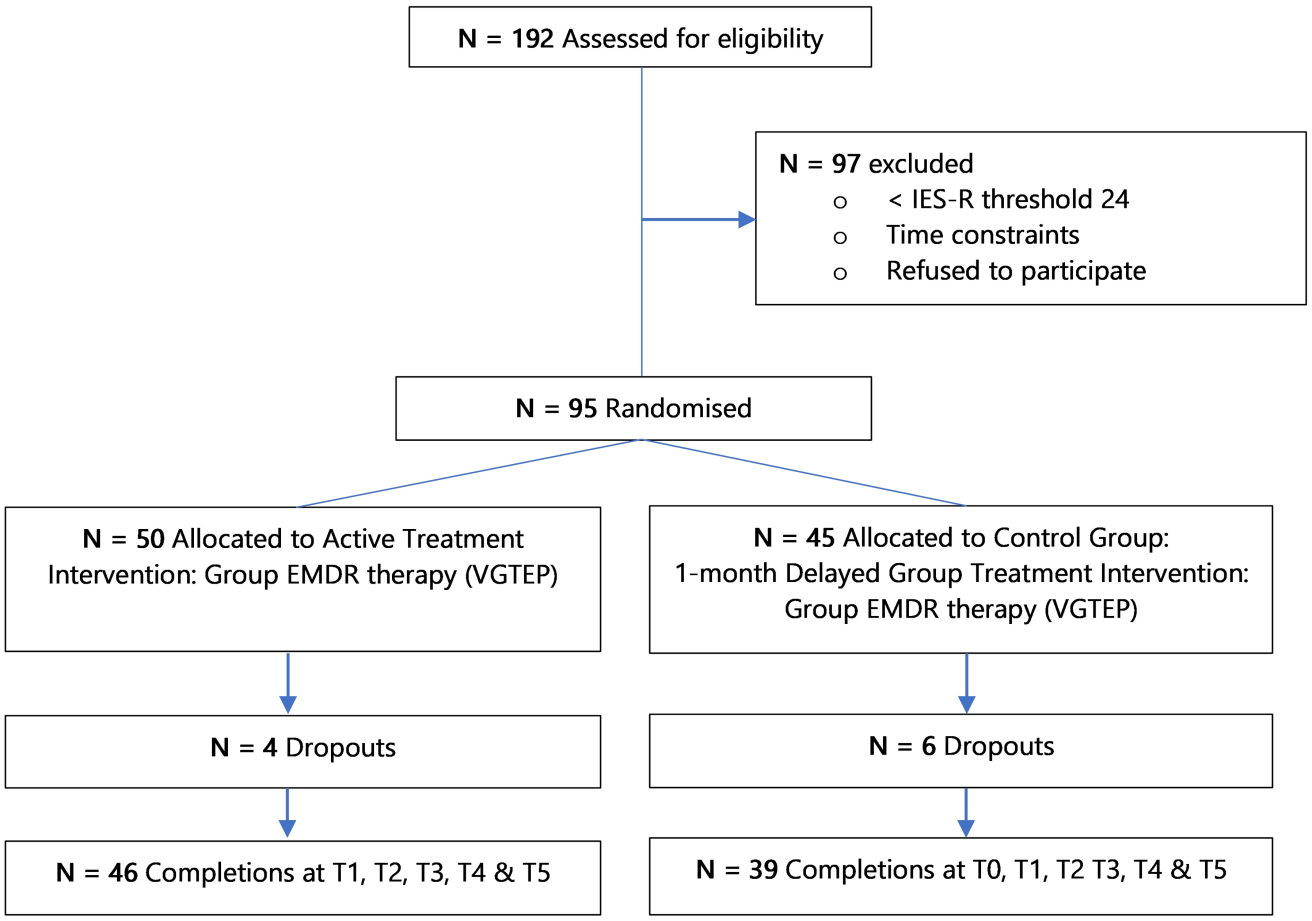
Flow of participants through the research clinical trial.

In determining the sample size^1^ was used setting α = 0.05, β = 0.2. A power calculation indicated an initial sample size estimation of *N* = 72 with *N* = 36 in each arm of the study. This figure allowed for a 10% drop-out rate. Randomization done through sequence generation using a computer algorithm allocating each research participant a unique client number (UCN) to ensure anonymity to the clinical team. Figure 2 highlights *N* = 192 clients assessed for eligibility and *N* = 97 individuals excluded from the study as their IES-R scores were < 24, leaving a total of *N* = 95 subject to randomization.

### Measures

The clinical trial used the following measures:

o International Trauma Questionnaire (ITQ) – an 18 question, self-reporting measure focused on both PTSD and Complex PTSD, consistent with ICD-11 (Hyland et al., 2017; Cloitre et al., 2018; Redican et al., 2021).
o Generalized Anxiety Disorder Assessment (GAD-7) which objectifies and assesses the degree of anxiety severity (Williams, 2014).
o Patient Health Questionnaire (PHQ-9) which objectifies and assesses the degree of depression severity (Kroenke et al., 2001).
o Subjective Unit of Disturbance/Distress (SUD) – a scale of 0 to 10, measuring the subjective intensity of disturbance or distress currently experienced by an individual (Wolpe, 1969).
o Moral Injury Event Scale (MIES) – 9-point self-reporting scale exploring perceived transgressions, betrayals, or violations of an individual’s moral code (Nash et al., 2013).
o Adverse Childhood Experience International Questionnaire (ACE-IQ) – tested at Pre point only with the intention to measure exposure to adverse childhood experiences (< 18 years old).
o Benevolent Childhood Experiences Score (BCEs) (Narayan et al., 2017) – tested at Pre point only: this is a measure of exposure to benevolent factors that occurred in childhood that may impact on resilience and post-traumatic growth (< 18 years old).
o EQ-5D – generic measure of an individual’s health status. The EQ-5D is a preference based HRQL across five dimensions; mobility, self-care, usual activities, pain/discomfort, and anxiety/depression.

Measure evaluated after each VGTEP session: SUD.

Measures evaluated at T1 only: ACE-IQ and BCEs.

Measures evaluated at T0, T1, T2, T3, T4, and T5: ITQ, GAD-7, PHQ-9, EQ-5D.

Measures evaluated at T1 and T5 only: MIES.

All psychometrics were sent out electronically to all research participants *via* the Trauma Response Network Ireland Administrators and was collected independent to the research team.

### Treatment intervention

Once research participants met the study’s inclusion criteria the TRN administrative team randomly assigned participants using a random allocation software package to either the active group Immediate Treatment Group) or a 1-month Delayed Treatment Group. Each group intervention involved a 1-week block of intensive treatment consisting of four, two hours sessions of Group EMDR therapy, delivered as a remote treatment intervention. The platform used for the VGTEP sessions was ZOOM Enterprise version 5.9.1. The TRN Ireland administrative team emailed a ZOOM link for each of the treatment sessions – evenings of Monday, Wednesday, and Thursday, and Saturday morning. At the commencement of each treatment session research participants were instructed to only use the unique client number as a means of identification, and not their name. Clinical members of the research teams had no means of identifying any of the research participants. Participants were required to attend all four treatment sessions. Each session lasted approximately 2 h. Each group consisted of six participants who remained as a group for the duration of the treatment intervention. In addition, the VGTEP session lead, and the emotional protection worker (EPW), were also randomly allocated to the treatment sessions. As per the research protocol, session leads and EPWs introduced themselves at the start of each VGTEP treatment session, and importantly, however, the research participants revealed no information about themselves. Additionally, it was emphasized to the research participants that the VGTEP intervention required no disclosure of anything about the trauma memory – consistent with the EMDR Blind 2 therapist protocol. Clinical Leads and Emotional Protection workers were also randomly allocated to the VGTEP treatment intervention ensuring therapist rotation by the TRN Administrative team.

The EMDR Group Treatment Intervention delivered as video-conference psychotherapy (VGTEP) consisted of seven steps: 1. Trauma regulation exercise, 2. Trauma target selection, 3. Anchor to a past resource, 4. Identification of a future belief, 5. Trauma confrontation of the target trauma memory, 6. Enhancement of resilience and post-trauma growth, and 7. Closure grounding exercise. Dual attention, and bilateral stimulation, a distinct element of EMDR therapy, are used in steps 2 and 5. Step 5 addresses three of the significant points of disturbance relating to the target trauma memory. Trauma processing within the confrontation stage of VGTEP involves 27 occasions of focus on the trauma target material.

Each VGTEP session was digitally recorded and subjected to treatment fidelity checks carried out by an independent EMDR Europe Accredited Consultant/Clinical Supervisor. When necessary, the Emotional Protection worker utilized the breakout room within ZOOM to manage any participant if they needed I:I support or guidance. Before the commencement of the trial, training was provided for both the VGTEP clinical leads and Emotional Protection workers, this ensured familiarity with the clinical trial, treatment fidelity, triage and risk assessment procedures, welfare checks, team support, and clinical and research supervision. Mirabilis Health Institute, Belfast, Northern Ireland, provided onward referral and Consultant Medical Supervision for the clinical trial.

Regarding treatment fidelity and integrity a treatment protocol manual was created for the clinical trial, based on the Elan Shapiro GTEP Training Manual – version 9 (unpublished manual). Each treatment session was video recorded and was independently fidelity checked by an international expert in the Group Traumatic Episode Protocol (GTEP). Additionally, all psychometrics were acquired independently to the research team, with the entire data set only handed over to the team for data analysis once all the 6-month follow-up data had been obtained.

### Data analysis

Data Analysis used the Jamovi (version 2.3.21.0) statistical software package. Descriptive data analyzed used either independent sample t-tests or fischer exact test. For further descriptive statistics, mean ±, standard deviation (±SD) for numerical variables and percentage (%) for categorical data. Tests for normality and homogeneity of variance determined no violations, therefore repeated measures RM-ANOVA analyzed each variable at T0, T1, T2, T3, T4, and T5 time points. The rationale for utilizing RM-ANOVAS is that they have been frequently used in various RCT studies testing EMDR therapy (de Roos et al., 2011; Jarero et al., 2018; Osorio, 2018; Ostacoli et al., 2018; Yurtsever et al., 2018). Skewness and kurtosis ensured normality of the ACEs and BCEs scores, as well as Pearson r to determine correlation coefficients. The *p* value of <0.05 considered significant. Using RM-ANOVA models Eta squared η^2^ is used as an indication of effect size (Olejnik and Algina, 2003).

## Results

Within the research trial, 64 VGTEP treatment sessions were carried out during two periods of government lockdown during the COVID-19 pandemic. This equated to approximately 128 h of treatment intervention, including both active (*N* = 46) and control (*N* = 39) groups. Per the therapist rotation model, *N* = 11 EMDR therapists carried out the 64 treatment interventions, and *N* = 26 Emotional Protection Team members were utilized. VGTEP Therapists, Emotional Protection Workers, and research participants were blinded in the intervention.

Metric points conducted at T0 (control), and then T1 (pre-treatment), T2 (post—treatment), T3 (1-mth FU), T4 (3-mth FU), and T5 (6-mth FU). Table 3 highlights *N* = 85 completed the study up to, and including, T5.

**Table 3:** Descriptive data.

Characteristics	Active: group EMDR (*n* = 46)	Control: delayed group EMDR (*n* = 39)	Total sample (*n* = 85)	*p* value
Age, mean (SD)	46.4 (9.78)	45.5 (11.9)	45.99 (10.72)	0.340^1^
Gender no				0.792^2^
- Male	9	9	18	
- Female	37	30	67	
Location				0.384^2^
- All Ireland	22	23	45	
- International	24	16	40	
Occupation category				0.787^2^
- Frontline Healthcare	35	33	68	
- First Responders/ Emergency Workers	10	7	17	
Trauma Onset				0.014^2 *^
- A: less that 6-months	2	10	12	
- B: 6–12 months	41	27	68	
- C: 1 year +	3	2	5	
ACE scores mean, (SD)	2.78 (2.46)	3.36 (2.49)	3.05 (2.47)	0.856^1^
BCE scores mean (SD)	8.09 (2.14)	8.00 (1.84)	8.05 (1.99)	0.421^1^

^1^Independent samples *t*-test. ^2^Fischer exact test. *sig *p* < 0.05.

**Figure 3:**
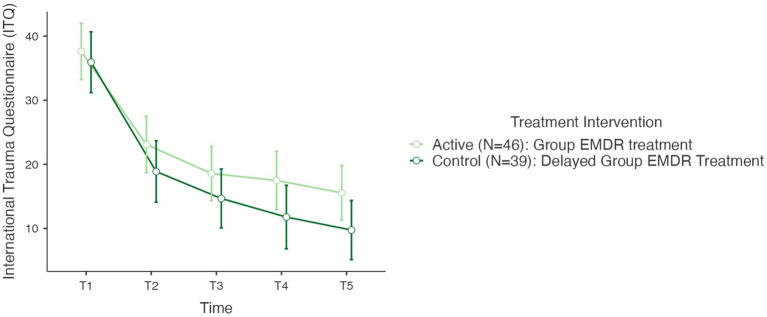
International trauma questionnaire (ITQ) T1–T5 active and control group (*N*=85) @ *p* < 0,001. T1, pre; T2, post; T3, 1-mth FU; T4, 3-mth FU; T5, 6-MTH FU.

**Figure 4:**
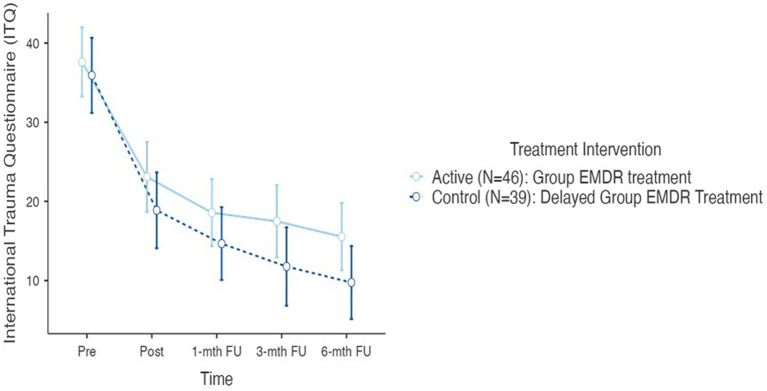
International trauma questionnaire (ITQ) T1–T5 active and control (delayed) groups (*N*=85) @ *p* < 0.001.

In testing Hypothesis 1: There is no difference between active and control (delayed) groups regarding trauma sequelae—measured with the International Trauma Questionnaire (ITQ) when comparing T1, T2, T3, T4, and T5. As mentioned earlier results revealed no statistical change between T0 and T1 for the control group, therefore, Figure 3 highlights the descriptive results for the complete cohort *N* = 85 for periods T1, T2, T3, T4, and T5. A repeated measures RM-ANOVA conducted to compare the impact of the Virtual GTEP intervention on trauma symptoms using the International Trauma Questionnaire (ITQ) evaluated at T0 (control group), T1 – pre, T2 – post, T3–1-mth FU, T4–3-mth FU, and T5–6-mth FU. For the control (delayed) group there was no statistical difference between T0 and T1. Results, highlighted in Figure 3, demonstrated a significant difference in the reduction of the ITQ scores following the treatment intervention over time *F*(4–332) = 106.84, *p* < 0.001. *Post Hoc* comparisons of the ITQ demonstrated a significant difference between T1 pre (mean 36.8, SD 14.8) and T2 post (21.2, 15.1) (t11.58) = 15.68, *p* < 0.001). This was also seen between T1 pre and T3 1-mth FU (16.8; 14.5) (t13.77) = 20.06, *p* < 0.001; T1 pre and T4 3-mth FU (14.9, 15.7) (t14.02) = 21.98, *p* < 0.001; and T1 pre and T5 6-mth FU (12.9, 14.7) (t15.11) = 24.12, *p* < 0.001. Finally, there was a significant difference between T2 post and T4 3-mth FU d = 0.41, and T5 6-mth FU d. Figure 4 highlights the T1 and T5 scores, including outliers in the T5 dataset.

Concerning the ITQ, *post hoc* comparison determined no statistically significant difference between the active and the controlled (delayed) treatment interventions. In determining the overall effect size of the treatment intervention regarding the ITQ, as a repeated measures ANOVA used Lakens (2013) cites Cohen (1992) in providing a benchmark for η^2^ as an indication of effect size: η^2^ = 0.01 (small), η^2^ = 0.06 (medium), and η^2^ = 0.14 (large). Results of the ITQ treatment effect size calculated at η^2^ = 0.420 indicating a large treatment effect.

**Figure 5:**
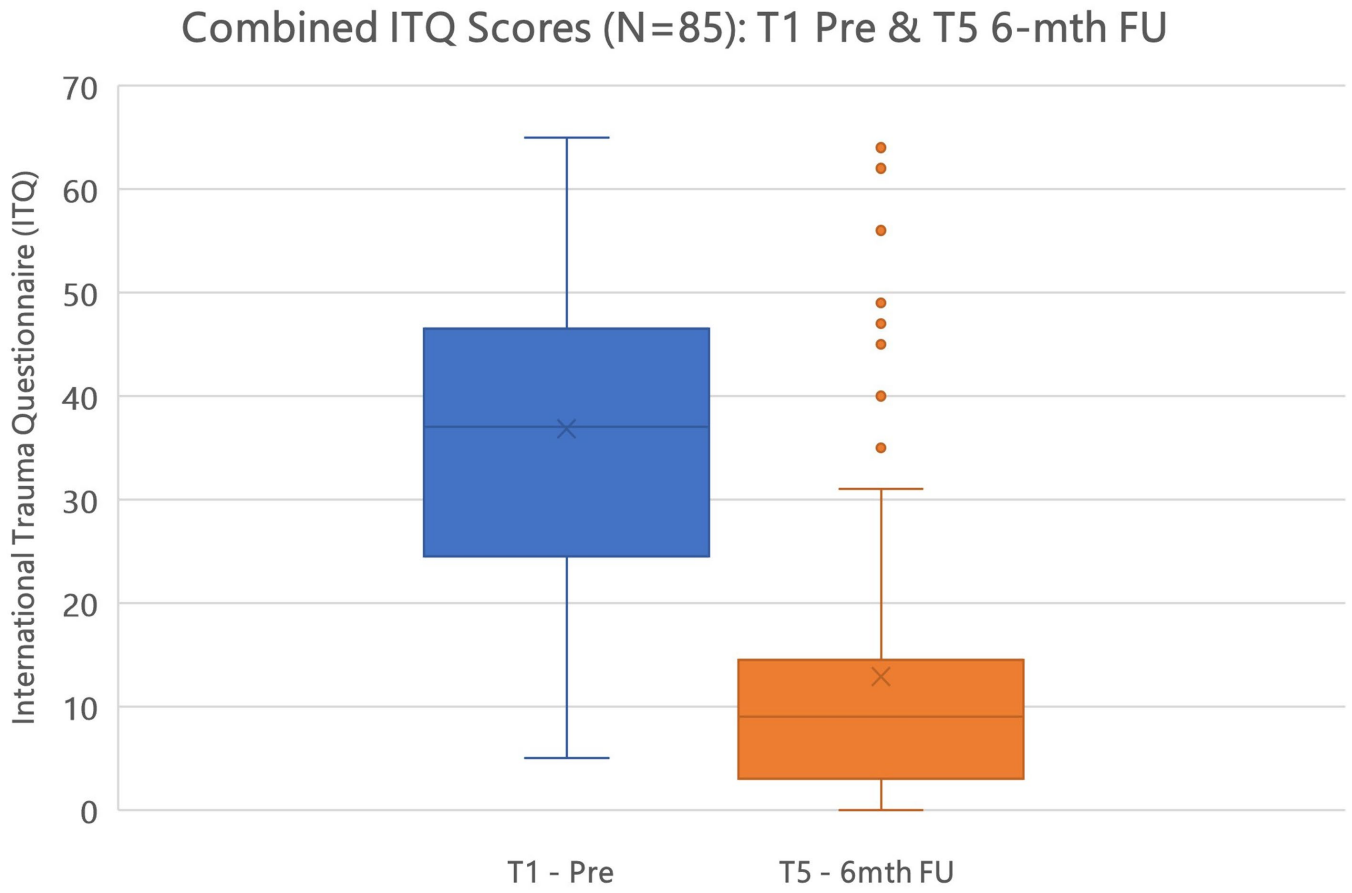
Combined ITQ T1 pre and T5 6-mth FU scores active and control (delayed) groups (*N*=85) @ *p* < 0.001.

During the VGTEP session there are stages where the participants subjective unit of disturbance is assessed. This subjective measure ascertained in steps 1, 2, 5, and 6 of the VGTEP protocol, however, per the research protocol SUD measures obtained pre and post step 1, and at points step 2 and step 6. The rationale for this is to consider changes in subjective distress during both the trauma regulation (step 1) and trauma confrontation (steps 2–6) parts of the VGTEP protocol. Figure 5 highlights the alterations in the SUD during the trauma confrontation stage of the *N* = 340 participant episodes of VGTEP. The SUD treatment effect size calculated at η^2^ = 0.295, *p* < 0.001, indicating a large treatment effect.

Table 4 demonstrates alterations in the SUDs phenomenology (mean, SD, median) during the entire VGTEP session including both trauma regulation and confrontation stages. Results suggest that the Trauma Regulation element has a distinct treatment effect overall (η^2^ = 0.189, *p* < 0.001), and therefore is of clinical relevance and benefit.

**Table 4:** Descriptive data measuring Subjective unit of disturbance during VGTEP session (*N* = 340).

	Treatment group	VGTEP step 1 pre	VGTEP step 1 post	VGTEP step 2 (pre)	VGTEP step 6 (post)
Mean (SD)	Active (*N* = 46)	5.06 (2.14)	2.87 (2.23)	7.40 (1.98)	4.25 (2.64)
	Control (*N* = 39)	5.05 (2.34)	3.03 (2.00)	6.98 (1.92)	4.42 (2.18)
Median	Active (*N* = 46)	5	3	8	4
	Control (*N* = 39)	5	3	7	4

**Figure 6:**
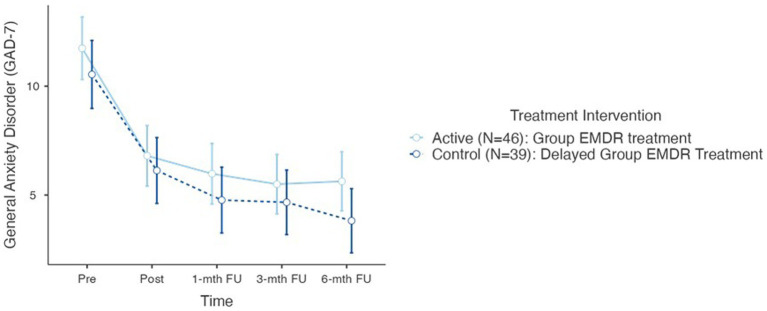
GAD-7 psychometric scores for active and control groups (total *N*=85).

**Figure 7:**
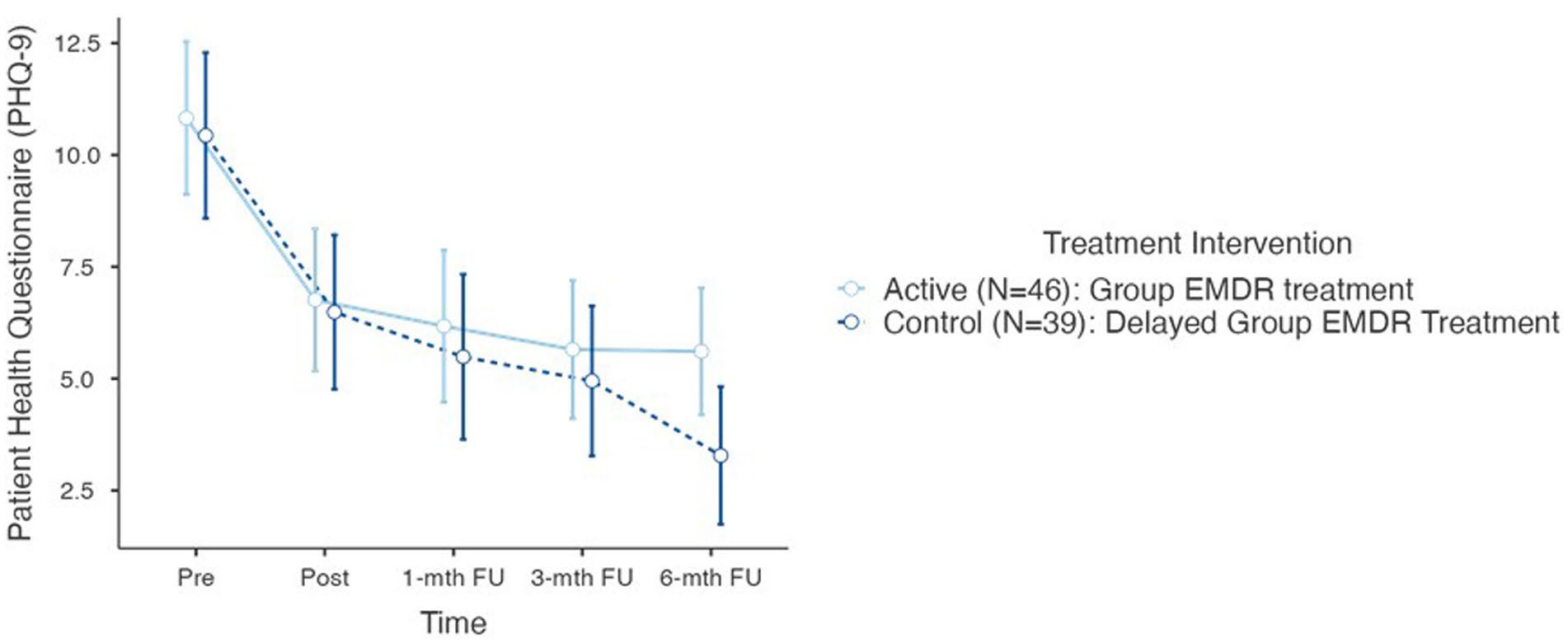
PHQ-9 psychometric scores for active and control groups (total *N*=85).

Figure 5 plots the alterations in SUD for both trauma regulation (Step 1 pre and post) and confrontation (Step 2 and 6). Results also highlight the range scores in that within each VGTEP session SUD scores decreased, increased, and flatlined, however, the general trend was a statistically significant (*p* < 0.001) decrease (Figures 6, 7).

For the VGTEP SUD scores for the individual (*N* = 340) sessions results demonstrated a significant reduction in both groups over time for both trauma regulation (Step 1) and trauma confrontation (Step 2 and 6) with no statistical difference between the active and control (delayed) groups. The overall effect size for the trauma regulation piece @ *p* < 0.001, with an effect size calculated at η^2^ = 0.189, and trauma confrontation η^2^ = 0.295.

In summary, when reviewing hypothesis 1 the null is supported – no difference was observed between the active and control groups indicating that the VGTEP treatment intervention was effective for both groups.

Testing Hypothesis 2: There is no difference between active and control groups regarding co-morbid sequelae – measured with the GAD-7 and the PHQ-9 at T1, T2, T3, T4, and T5.

Further repeated measures conducted to compare the impact of the trauma intervention on both the Generalized Anxiety Disorder Assessment (GAD-7) and the Patient Health Questionnaire (PHQ-9), evaluated at T0 (control group), T1 – pre, T2 – post, T3–1-mth FU, T4–3-mth FU, and T5–6-mth FU. Results determined no statistical difference between the two treatment group interventions for either the GAD-7 or the PHQ-9.

For the GAD-7 results demonstrated a significant reduction in both groups over time *F*(5–190) = 42.3, *p* < 0.001. There was no difference between T0 and T1. However, *Post Hoc* comparisons of the GAD-7 demonstrated significant difference between T1 pre (11.2, 4.91) and T2 post (6.49, 4.73) (*t* = 6.22) = 4.41, *p* < 0.001; T1 and T3 1-mth FU (5.42, 4.75) (*t* = 8.67) = 5.78, *p* < 0.001; T1 and T4 3-mth (5.12, 4.64) (*t* = 7.78) = 7.78, *p* < 0.001; T1 and T5 6-mth FU (4.73, 4.27) (*t* = 8.93) = 6.72, *p* < 0.001. In determining the overall effect size of the treatment intervention regarding the GAD-7, the overall effect size calculated as η^2^ = 0.202 indicating a large treatment effect.

For the Patient Health Questionnaire (PHQ-9) results also demonstrated a significant reduction in both groups over time *F*(5–190) = 43.3, *p* < 0.001. Furthermore, like the ITQ and GAD-7, there was no statistical difference between T0 and T1. However, *post hoc* comparison of the PHQ-9 demonstrated significant differences between T1 pre (11.7, 5.68) and T2 post (6.64, 5.79) (*t* = 6.30) = 3.95, *p* < 0.001, *d* = 0.71; T1 and T3 1-mth FU (5.86, 5.77) (*t* = 6.22) = 4.95, *p* < 0.001, *d* = 0.82; T1 and T4 3-mth FU (5.33, 5.24) (*t* = 6.39) = 5.49, *p* < 0.001; and T1 and T5 6-mth FU (4.54, 4.94) (*t* = 8.89) = 7.15, *p* < 0.001. In determining the overall effect size of the treatment intervention regarding the GAD-7, the overall effect size calculated as η^2^ = 0.136 which is on the threshold of a large treatment effect.

Regarding hypothesis 2 the null hypothesis is accepted – no difference was observed between the active and control groups demonstrating that the VGTEP intervention was influential in the treatment of co-morbid features of anxiety and depression.

*Hypothesis 3*: Treating the trauma sequelae will reduce the level of moral injury in active and control groups.

**Figure 8:**
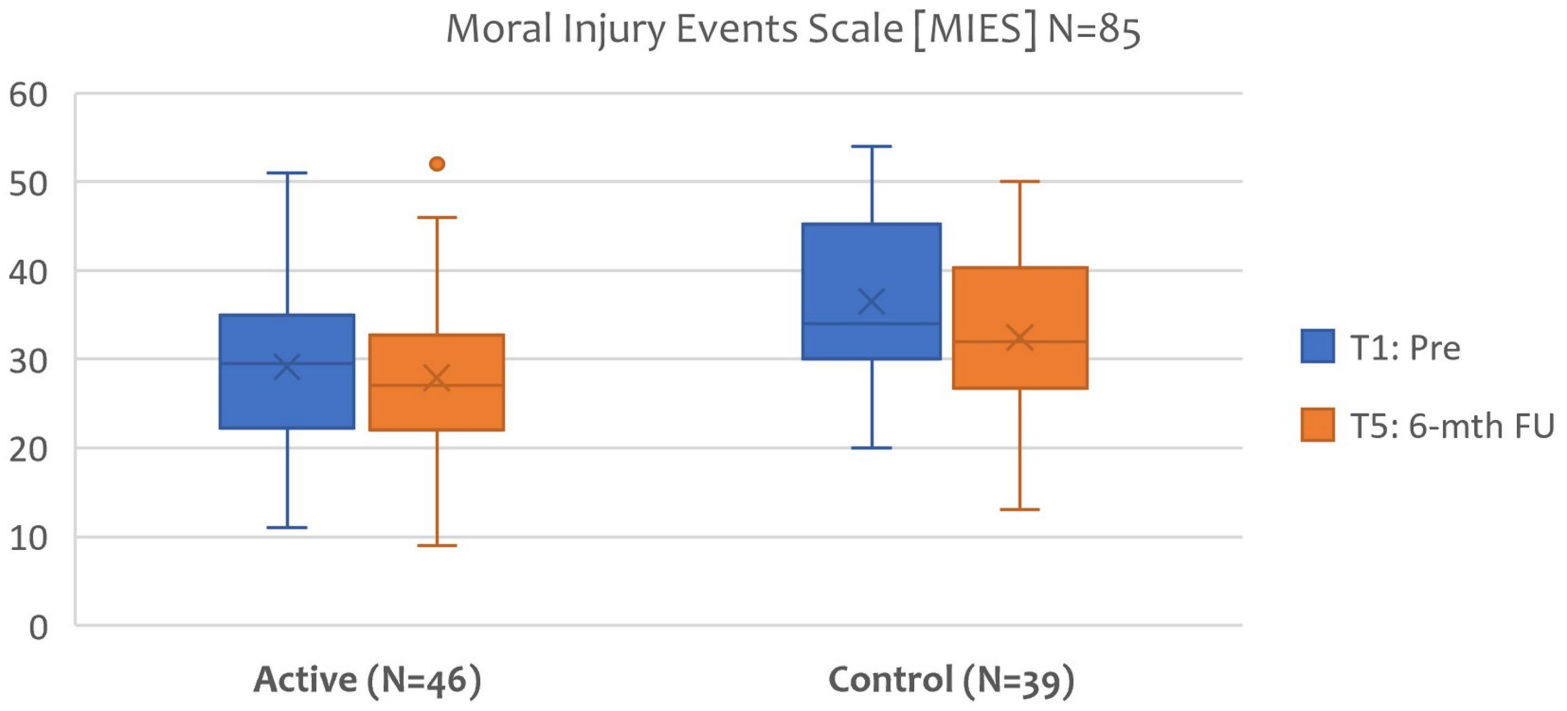
Moral injury events scale (MIES): T1 Pre and T5 6-mth FU (*N*=85)..

The Moral Injury Events Scale (MIES) was selected for this study as it has been adapted and applied within civilian research, has the advantage of measuring both events and symptoms, and, as a psychometric, is brief and straightforward in wording (Nash et al., 2013; Koenig et al., 2019). Although the MIES has a three-factor structure: moral violations either perpetrated or witnessed by the individual, and betrayal experiences results at this stage only focused on total scores. Further analysis will be presented in a future paper. Figure 8 demonstrate no treatment effect between T1 pre and T5 6-month FU, measured by the MIES, of the VGTEP intervention in both active and control groups.

Regarding hypothesis 3, the results do not support this hypothesis. Although the VGTEP intervention effectively treated trauma, anxiety, and depression symptoms, results demonstrate no discernible treatment effect on moral injury.

*Hypothesis 4*: Using Trauma-Focused Therapist rotation will demonstrate no change in clinical diagnosis between T1 and T5, for both active and control groups.

Regarding the diagnoses of PTSD and Complex PTSD, using the ITQ as a diagnostic indicator, Table 5 highlights the impact of the VGTEP treatment intervention at post-treatment (*N* = 85). At T1 *N* = 24 had sub-clinical PTSD, *N* = 28 PTSD, and *N* = 33 Complex PTSD. Of the research participants with sub-clinical PTSD at the start of the treatment intervention results indicated that 95.83% remained sub-clinical at T5 6-month FU, with 4.17% meeting the criterion for PTSD. Those meeting the criterion for Complex PTSD results suggest a 72.73% improvement to sub-clinical, however, 21.21% showed limited improvement from the VGTEP treatment intervention.

**Table 5:** Changes in clinical diagnosis following VGTEP intervention using the ITQ at T1 and T5 (*N* = 85).

Initial diagnosis @ T1 pre-treatment	No.	Final diagnosis @ T5 6-mth FU	No.
o Sub-clinical PTSD^1^	24	Sub-clinical PTSD	23
		PTSD	1
		Complex PTSD	0
o PTSD	28	Sub-clinical PTSD	28
		PTSD	0
		Complex PTSD	0
o Complex PTSD	33	Sub-clinical PTSD	24
		PTSD	2
		Complex PTSD	7

1 Sub-clinical IES-R score of 24-32 (clinical concern).

Despite using a therapist rotation model within the clinical trial results highlight significant alterations in diagnosis at T5 6-mth FU. The VGTEP interventions had marked efficacy with both the sub-clinical and the PTSD research participants. In the sub-category, only one research participant was referred for additional help and support. With a dropout rate of 10.53% from the clinical trial, and 11.76% finding the treatment intervention insufficient, the overall recovery rate from the VGTEP intervention is estimated at 77.71%. Results confirm that hypothesis 4 is not supported – a favorable recovery rate was achieved using a therapist rotation model.

*Hypothesis 5*: There is no difference between active and control groups regarding quality of life using the EQ-5D – a generic measure of an individual’s health status and quality of life.

**Figure 9:**
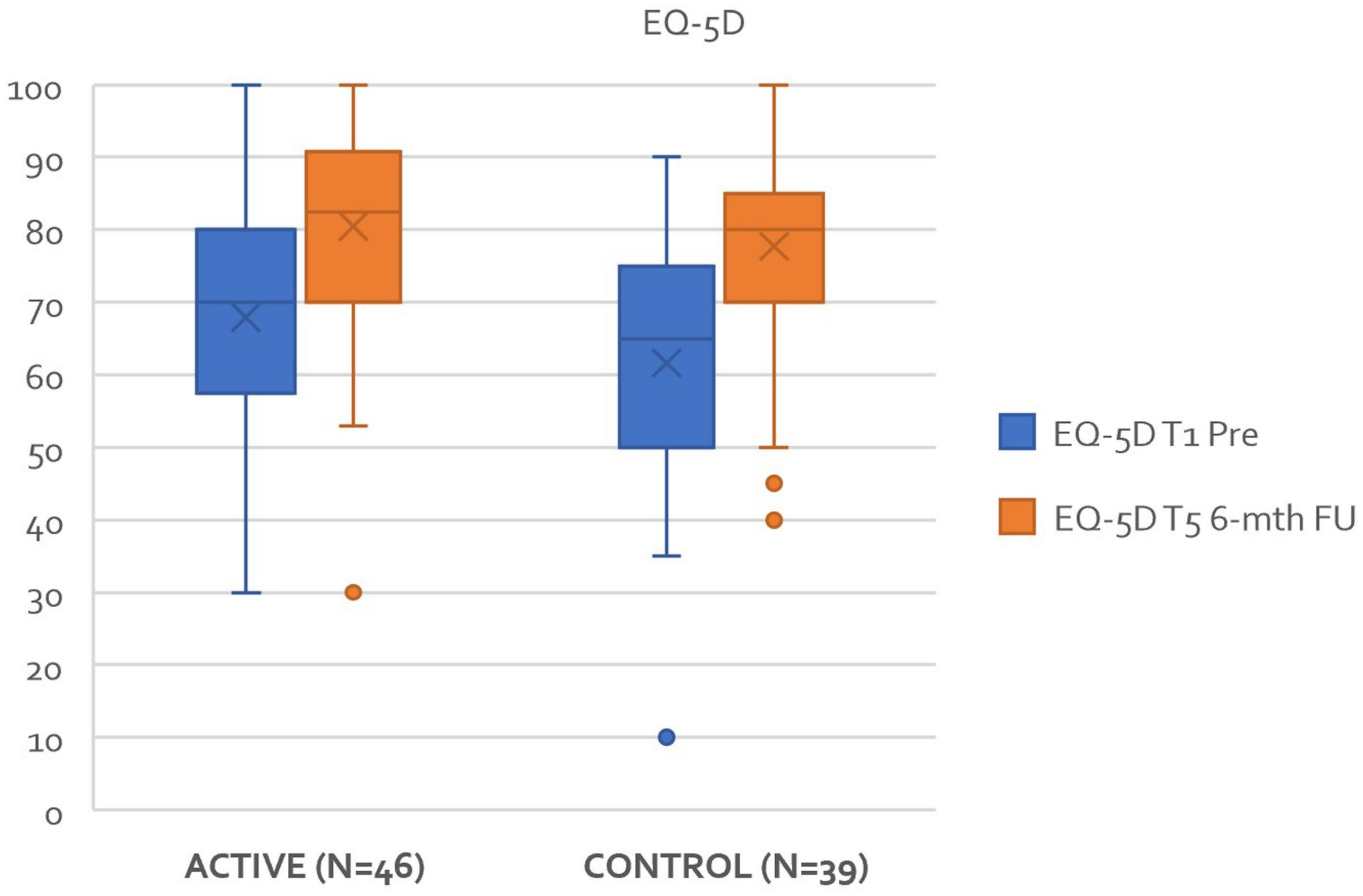
Improvements in EQ-5D between T1 and T5 active and control groups.

Figure 9 highlights that for the EQ-5D results demonstrated a significant reduction in both groups over time *F*(1–83) = 48.8, *p* < 0.001. *Post Hoc* comparisons of the EQ-5D demonstrated a significant difference between T1 pre (65.02, 17.99) and T5 (79.19, 14.84) t(−6.99) = −14.3, *p* < 0.001. In determining the overall effect size of the treatment intervention regarding the EQ-5D, calculated as η^2^ = 0.159 indicating a large treatment effect. There was not statistical difference between each of the groups. A more in-depth review of the health economic data will follow in a later paper.

*Hypothesis 6*: A correlation exists, within the target population, between Adverse Childhood Experiences (ACEs) and Benevolent Childhood Experiences (BCEs) regarding evidence supporting resilience and posttraumatic growth.

A Pearson correlation coefficient was computed to assess the linear relationship between Adverse Childhood Experiences (ACEs) and Benevolent Childhood Experiences (BCEs). Results revealed a moderate, negative correlation between the two r = −0.312, **p = <0.01 as highlighted in Table 6. Bivariate analysis of either ACEs or BCEs revelated no other linear relationships with the ITQ, GAD-7, PHQ-9, MIES, or EQ-5D. Results indicate that the VGTEP treatment intervention was effective regardless of a research participants ACE or BCE scores.

**Table 6:** Correlation matrix between ACEs and BCEs.

		Total ACE Score	Total BCE score
Total ACE Score	Pearson’s r	—	
	*p* value	—	
Total BCE score	Pearson’s r	−0.312**	—
	*p* value	0.004	—

*p < 0.05, **p < 0.01.

Table 7 highlights qualitative data related to the research participants which focused on the drop-outs from the study. Each participant was followed-up and offered further support and intervention. As their withdrawal impacted upon the T2 – T5 psychometric data their data removed from the overall data set. Although a 10.58% drop-out rate is low it is also reasonable to assume that not every client, once they have direct experience of working in this way, will find it useful.

**Table 7:** Qualitative factors which impacted upon T2–T5 psychometric data.

Theme	Number and %
Referred for follow-up care after T2	9 (10.58%)
Struggled with continued post-COVID symptoms/ complications (long COVID) @ T5	5 (5.89%)
Additional post-trauma experiences not COVID-19 related post T2	4 (4.71%)

**Figure 10:**
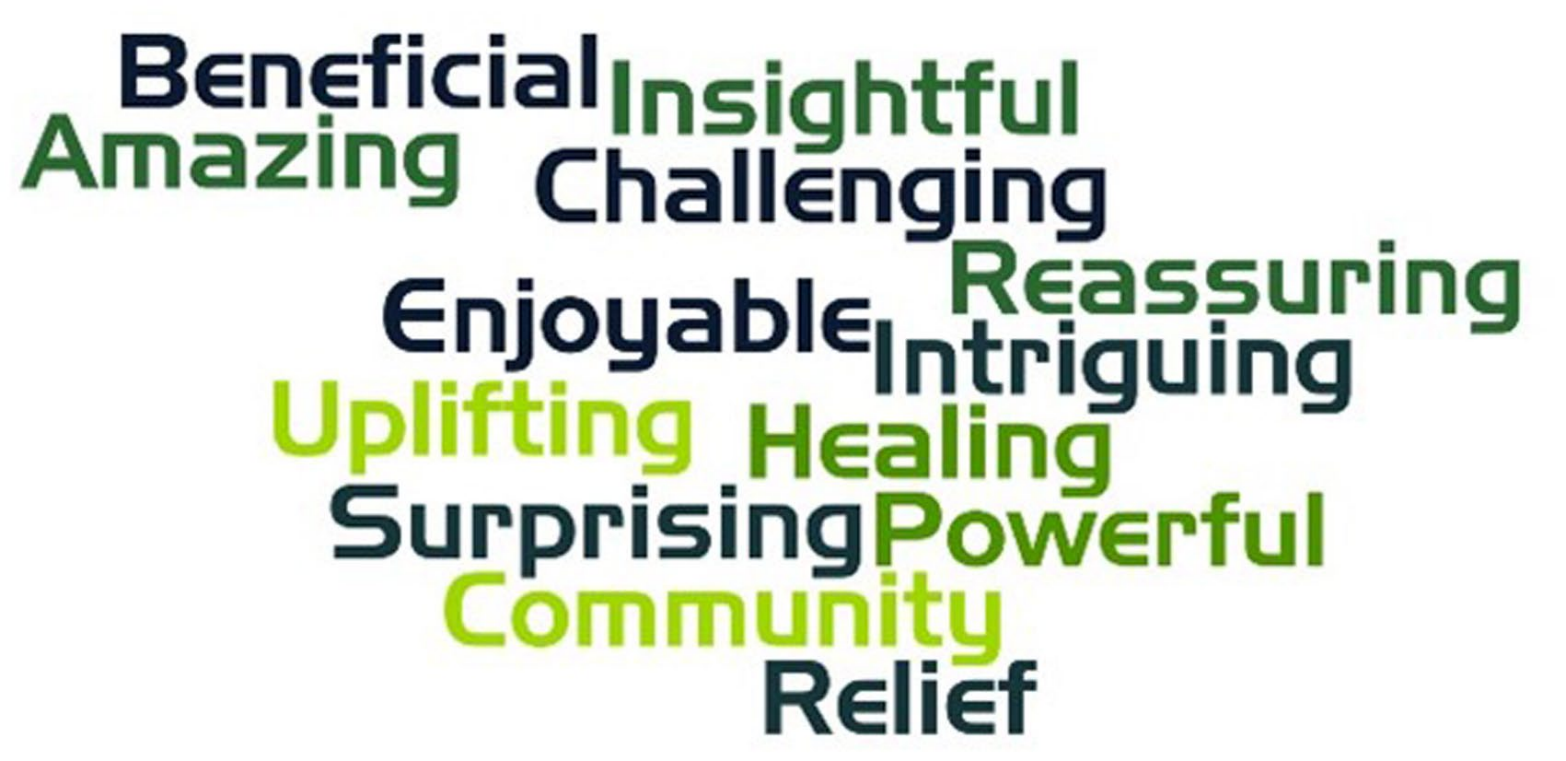
Primary themes related to the qualitative feedback received from research participants post T5 (6-mth FU/end of treatment).

At T5 6-month FU, every research participant contacted to provide brief feedback about their lived experience of undergoing the VGTEP intervention. A further research paper will explore this in more detail. However, Figure 10 highlights the primary response which clustered around 13 themes. The overwhelming feedback appeared to suggest the VGTEP intervention to have been effective, helpful, and timely.

## Discussion

Undertaking any large-scale, early intervention clinical trial is always difficult and present significant logistical challenges. Doing so during periods of Government-Imposed lockdown was also immensely demanding. The rationale for the study was to carry out a multi-component research project which focused on an important population at the frontline of the COVID-19 response. The realities of the COVID-19 pandemic, and carrying out clinical research, meant that everyone involved – the research participants, research team, and clinicians were all exposed to the ‘lived experience’ realities of COVID-19. Therefore, a reasonable conclusion is that the universality level for this whole study was extraordinarily high. As highlighted within the literature review, defining early intervention is not straight forward. Considering the ongoing nature of the pandemic for the entire duration of this RCT there is an argument cogent argument for this qualifying as an early intervention study.

An important reality of the current time was that lack of availability of trauma-informed early intervention for frontline emergency workers paradoxically assisted recruitment for our study. The consequence of this resulted in most research participants highly motivated, and appreciative to being included. Despite the eventual recruitment, a larger sample would have further enhanced the study’s findings. However, as this was an unfunded study, it was essential to make the limited resources we had stretch as far as possible.

The headline results from this RCT suggest that EMDR therapy, delivered remotely through a virtual platform was effective in the treatment of both PTSD and complex PTSD. This is an important finding considering the critical elements of group treatment, remote intervention, intensive delivery, and therapist rotation. When demand outstrips supply regarding access to trauma interventions research supporting the benefits of group treatment suggest significant resource benefits. Further research is needed to explore the potential benefits of this in relation to task-shifting.

However, based upon this data set, results do not support its efficacy as a treatment for Moral Injury. Although results are favorable regarding the ITQ, GAD-7, PHQ-9, SUD, and EQ-5D, demonstrating that the VGTEP intervention appeared relevant to the research population under investigation, results also suggest that the intervention was well tolerated, potentially safe, and effective as a treatment intervention. Intriguingly, there was no demonstrable difference between active and control (delayed) groups. Despite promising results, caution also needs to be exercised. The absence of another trauma-focused treatment intervention as a realistic comparator suggests that more research is required to ascertain a more reliable efficiency indicator. For example, future early intervention should consider comparing VGTEP with another Trauma-focused CBT Group Intervention.

Overall, the dropout rate for the study was just below 11%. There are two ways of looking at this; firstly, this level is particularly favorable to the context in which the research study operated – during a global pandemic. A contrary perspective relates to the tolerability of any psychological treatment intervention. There will always be dropouts related to factors unrelated to the study intervention – this was certainly the case for some but by no means all. However, following up on those that did drop out and relate to the treatment intervention highlighted that for some, it was too intense, too powerful, and too overwhelming, bearing in mind everything else going on in people’s lives at the time.

There are four other factors to consider. Firstly, treatment was delivered as video-conference psychotherapy. Overall results suggest that this was generally well tolerated. Although there were occasional technical glitches, fortunately, these were relatively infrequent. Indeed, the ZOOM platform performed robustly for much of the study—Secondly, the intense delivery of the treatment within 1 week. Again, results suggest that this was well tolerated, although admittedly, it does require a significant commitment from research participants. Further research needs to explore the potential health economic benefits of delivering trauma treatment in this manner. Thirdly, the VGTEP intervention involves non-disclosure of any trauma material. This aspect has advantages and disadvantages. Advantages include a greater willingness to work on traumatic material without the need for disclosure. This maximizes both power and control for the research participants. If trauma memories involve shame, fear of retribution, or a shattering of assumptive networks then there are clear benefits to non-disclosure. Disadvantages include a difficulty in demonstrating cause and effect. Although research participants were invited to work with recent material there was no means to accurately monitor this. This both a strength and limitation to this study. That said, previous research demonstrates qqclear benefits from working with non-disclosed trauma material (Farrell et al., 2020, 2022). Non-disclosure of trauma material does have another distinct advantage as non-disclosure of trauma material potentially reduces the risk of vicarious traumatization of therapists. However, more research is needed to explore this aspect further to ascertain if there is veracity in this argument.

The study’s results shed interesting light on the therapist rotation model. Instinctively, therapist rotation seems counterintuitive, considering the therapist relationship critical to outcome. Results from this study run counter to this narrative. Why is therapist rotation effective? There are several reasons to consider. The VGTEP intervention is highly structured – there are distinct benefits from this, reassurance, familiarity, containment, etc., however, the intervention is relatively passive. This argument may provide a rationale for why VGTEP was effective for PTSD/C-PTSD, but not Moral Injury (MI), is that MI requires more active engagement. This would require further investigation and study, and if proven correct, would necessitate further adaptation in EMDR group interventions.

The GTEP protocol involves 27 episodes of trauma confrontation and requires further research and investigation, particularly into its distinctive parts – which are essential, which not? Results from this study demonstrate an overall treatment effect; however, further dismantling studies need to explore whether there might be more effective or efficient means of achieving similar or better results.

Results from Table 5 are intriguing. Within the first category 28% were sub-clinical PTSD, and post-treatment 96% remained in this category. Could this potentially suggest that early intervention Group EMDR prevented the onset of PTSD? Further research is needed to explore this, however, even though results from this study are promising caution against over-reach is important. Of further interest relate to the PTSD and Complex PTSD data which suggest the favorability of the Group EMDR intervention. Again, these results are cautiously promising, however, further research is needed.

Roberts et al. (2019) conclude little evidence that most multiple-session trauma treatment interventions have little impact on primary outcome measures. However, our study’s results do not concur with this perspective. There is, nonetheless, an important narrative to consider at this juncture – results from this study suggest a significant treatment effect for PTSD but not for moral injury. Why might this be the case? This is an intriguing question. A potential consideration relates to the ‘unknowingness’ of the trauma memory clients use to target and reprocess. The EMDR GTEP and VGTEP interventions are ostensibly passive treatments. Within conventional EMDR therapy, moral dimensions are often addressed more actively with the utilization of Cognitive Interweaves, which are much more bespoke to the client’s lived experiences. However, more research is needed to explore this further, but results suggest a potential need for modifying the existing group EMDR protocols to address moral dimensions of trauma more effectively.

Another critical point of note relates to Adverse and Benevolent Childhood Experiences. Results from this study parallel an earlier study (Farrell et al., 2022) demonstrating a trauma treatment effect irrespective of either individual or collective ACEs or BCEs. Although results show a moderate, negative correlation between ACEs and BCEs, this certainly needs further research and exploration. The evidence from this study suggests that adverse memories process in EMDR therapy regardless of the form the adversity takes. The way benevolent factors potentially mitigate does require further investigation, particularly utilizing the theoretical framework of Adaptive Information Processing – the model underpinning EMDR therapy.

We recognize that follow periods reported within this RCT study are short (Dumoulin et al., 2018), however we took the pragmatic decision to use three-and six-month timeframes for follow up given the relative urgency to provide online psychological support to frontline workers during the pandemic (Drissi et al., 2021). A recent systemic review (Wilson et al., 2018) suggests follow up periods of between 4 weeks and 6 months is not uncommon in EMDR intervention research. For example, in what appears to be the only trial of online EMDR (Lenferink et al., 2020), Spence et al., 2013 used a 3 month follow up period when examine the use of internet delivered EMDR. We are therefore content that the design chosen for the study is in line with other EMDR related randomized controlled trials, however, acknowledge that the longer-term maintenance of benefits from the intervention may not be fully understood.

A further, distinct limitation of the study relates to the issue of moral injury. Further research is needed to adapt EMDR group interventions more specifically more sufficiently for this important issue. Secondly, with the benefit of hindsight, incorporating more qualitative data surrounding the participants experience would have been useful. Presently, there is no absolute clarity surrounding agents of change other than from the psychometric data. Thirdly, it would have been useful to ascertain better insight into the target memories worked on by the research participants. This was always going to be challenging based the critical aspect of non-disclosure. A further aspect related to this would have been to determine if research participants may indeed have been willing to disclose their trauma targets. Fourthly, would be to consider if more detailed debriefing sessions after the VGTEP intervention may have further enhanced the study’s findings. Fifthly, as the research participants were ‘self-selecting’ this leads to the possibility of bias and ambiguity. By not capturing or clarifying motivation for being part of the study there is a fair degree of uncertainty about this aspect. Furthermore, a distinct limitation was not being able to ascertain which trauma targets were moral injury, or not. A final limitation relates to the sub-clinical PTSD participants. The data set suggests that many from this group remained ‘sub-clinical’ post-intervention, however, there is not means to determine cause and effect, that the intervention prevented the onset of PTSD. To ascertain this with more clarity further research is needed.

A contemporary debate within the EMDR therapy literature relates to the value of trauma regulation. The data presented in Figure 5 suggests treatment benefits for trauma regulation and trauma confrontation phases with VGTEP; however, the effect size is more significant for trauma confrontation. Nevertheless, results suggest the clear advantage of utilizing trauma regulation strategies within the VGTEP intervention, even if this provides a grounding, presentness, or even a sense of community-building. Further dismantling studies need to explore this aspect in more detail.

## Conclusion

The COVID-19 pandemic has presented significant challenges individually, collectively, and geopolitically. Ascertaining treatment interventions that can be delivered remotely safely, relevant to trauma populations, that are both effective and efficient, is paramount. In addition, the advantage of treatment given through video-conference platforms potentially increases availability and access. Critical to this relates to technical components, including availability, access, and functionality in making such treatment interventions viable on a fundamental level of scalability.

This research suggests the potential efficacy of the Group EMDR intervention; incorporating a therapist rotation model adds a contemporary facet that seems counterintuitive to the existing academic literature. In addressing the global burden of psychological trauma and the necessity for task-shifting, this study offers interesting findings to this debate.

The primary objective and rationale for this study were Frontline and Emergency colleagues working on the frontline of COVID-19 traumatized by their endeavors. This study contributes to the emerging knowledge base that Group EMDR therapy has something significant to contribute.

## Data availability statement

The datasets presented in this study can be found in online repositories. The names of the repository/repositories and accession number (s) can be found in the article/supplementary material.

## Ethics statement

The studies involving human participants were reviewed and approved by University of Worcester. The patients/participants provided their written informed consent to participate in this study.

## Author contributions

DF conceived the study, was the chief investigator for the entire project, the principal data analyst, and the primary author of this research manuscript. JM was responsible for project management. MK was responsible for secondary data analysis, senior leadership, and support to the project. LK, PM, IM, DM, TP, PP, CM, and IB were vital research team members and treatment interventions. ZZ was responsible for treatment fidelity. DF, LK, JM, PM, IM, DM, TP, and PP were all clinical leads regarding the VGTEP treatment intervention. All authors contributed to the article and approved the submitted version.

## Conflict of interest

The authors declare that the research was conducted in the absence of any commercial or financial relationships that could be construed as a potential conflict of interest.

## Publisher’s note

All claims expressed in this article are solely those of the authors and do not necessarily represent those of their affiliated organizations, or those of the publisher, the editors and the reviewers. Any product that may be evaluated in this article, or claim that may be made by its manufacturer, is not guaranteed or endorsed by the publisher.

## Footnotes

^1^http://www.openepi.com/Menu/OE_Menu.htm
